# Brace treatment is effective in idiopathic scoliosis over 45°: a prospective controlled study

**DOI:** 10.1186/1748-7161-8-S1-O35

**Published:** 2013-06-03

**Authors:** M Lusini, S Donzelli, F Zaina, S Negrini

**Affiliations:** 1 ISICO (Italian Scientific Spine Institute), Milan, Italy; 2 University of Brescia, Brescia, Italy; 3 IRCCS Don Gnocchi, Milan, Italy

## Background

Recently, positive results in bracing patients with idiopathic scoliosis (IS) above 45°, who refused surgery, have been presented in a retrospective study. Obviously, this can only give an efficacy analysis, since there is no control group, nor it is possible to know failures due to drop out.

## Aim

To present the prospective results of bracing patients affected by IS above 45° and still growing.

## Methods

Design is a prospective study including all IS patients with 45° or more, Risser 0-4, who had their first evaluation in our Institute from March 1st, 2003 to December 21st, 2010, and utterly deny any surgical intervention. Population: out of 59 patients, we excluded 2 still in treatment. 57 (11 males) were included, who at start of treatment had a mean of: 15.03±1.10 years of age, 52.5° Cobb (range 45-93°), and Risser 2 (0-4). Thirty-nine accepted a full-time brace treatment (BG) to try avoiding surgery, 18 served as controls (CG). Outcomes: radiographic and clinical data. Statistics: efficacy analysis in patients who completed treatment (38 in BG; 10 in CG; failures: surgery, progression 6° or more); intent-to-treat (failures also drop-outs). In CG, we had 8 patients not retrievable: they were considered as positive results (no progression or surgery) in the intent-to-treat analysis.

## Results

Efficacy analysis: Failures were 23.5% in BG, and 100% in CG. Intent-to-treat: failures were 20.5% in BG and 55.6% in CG. The Relative Risks (RR) of failure in CG were 4.3 (95% Interval Confidence - IC95: 3.6-4.9) and 2.7 (IC95 2.0-3.5) respectively in the two analyses (P<0.05). Conversely, the RRs of improvement were 1.6 (IC95 1.46-1.9) and 1.9 (IC95 1.6-2.2) (P<0.05). Patients joining treatment achieved a 10.4±10.7° Cobb improvement, an ATR reduction of 4.2±4.3°, and an aesthetic improvement of 2.8±1.9 points (TRACE). At the end of treatment, 24 patients were below 45°, including 6 of below 35°.

## Conclusions

Through this study, we conclude that conservative brace treatment (if correctly performed and managed) is a suitable alternative for those patients who reject any surgical intervention for idiopathic scoliosis above 45°. Indeed, this treatment largely provides excellent results, and in most cases stabilises the curve, with a subsequent improvement of the Cobb degrees and TRACE levels.
